# The International Medical Congress

**Published:** 1881-10

**Authors:** 


### THE INTERNATIONAL MEDICAL CONGRESS.

This body held its seventh annual meeting in London, August 2d—9th. The occasion brought together a large number of the leading medical men of the world. There were about four thousand present. Such an assemblage was probably never together before. It embraced a large amount of the leading medical talent of Great Britain, France, Germany, Austria, Spain, Italy, and the United States, and representatives from every other civilized country.

"Medicine was never more fully represented, or more publicly honored than in the great assemblage on the opening day, when the Royal Princes of the Teutonic and English empires, standing side by side on a platform, graced and dignified by the most representative and illustrious of physicians and surgeons of the world."

Side by side sat Paget, Jenner, Langenbeck, Pasteur, Virchow, Charcot, Donders, Austin, Flint, Pantalione, and many others of equal celebrity. The great hall was filled to repletion, all the seats were filled on both the gallery and main floor, and all standing room occupied.

The Congress was formally opened by England's Prince in a brief address, delivered in most excellent voice, enunciation and manner. It was received by enthusiastic demonstrations of appreciation. Sir James Paget made the opening address, and it proved to be one befitting the occasion. Its theme was inspired by the Congress, and it was delivered with an eloquence and fervor that was certainly stimulated by the scene before the speaker.

This address was followed by Virchow, Frazer and others. It is not practicable in the space allotted here to more than make reference to the opening exercises, except that daily meetings of the Congress were held in the afternoon, where interesting addresses were made and the general business matters of the Congress transacted.

The chief part of the work of the Congress was accomplished in the sections, of which there were fifteen, viz: Sec. 1st,

Anatomy; Sec. 2d, Physiology; Sec. 3d, Pathology; Sec. 4th, Medicine, *Subsection* Diseases of the Throat; Sec. 5th, Surgery; Sec. 6th, Obstetric Medicine and Surgery; Sec. 7th, Diseases of Children; Sec. 8th, Mental Diseases; Sec. 9th, Ophthalmology; Sec. 10th, Diseases of the Ear; Sec. 11th, Diseases of the Skin; Sec. 12th, Diseases of the Teeth; Sec. 13th, State Medicine; Sec. 14th, Military Surgery and Hygiene; Sec. 15th, Materia Medica and Pharmacology.

In order to give some idea of the work done in sections, the sessions of which were held in the forenoon of each day, the following from the report of the work of the Congress, given by the General Secretary, Mr. MacCormac, is presented, in which he says "that one hundred and nineteen meetings of sections had been held, at which four hundred and sixty-four written, and three hundred and sixty spoken communications had been made," thus making eight hundred and twenty-four communications. These would probably average thirty minutes each. From this statement it is apparant that an immense amount of work was done. The attention of each individual member was, of course, during the meetings confined to his own section.

Of the Dental section it can justly be said that the hours of its sessions were crowded with interesting matter; indeed, it was necessary to economize the time, and work very closely to the programme in order to get through at all in the time allotted. Two or three of the papers have already appeared in the Register, and others will follow; they will repay a thorough reading and careful study.

The social features were upon a grand scale, such as will never be forgotten by those who enjoyed them. The entertainments were far more numerous than could be attended by any one person. What with dinners, collations, converzationæ, garden and lawn parties, excursions, and the open doors of almost every place of public interest in and about London, one was almost in a state of bewilderment. The Dentists of London and vicinity entertained their guests right royally, and in such a manner as to strengthen the bonds of professional brotherhood, and give an impetus in this direction that will increase and grow stronger as time rolls on. The memory of that occasion, or occasions, will ever be a precious inheritance to those who were so fortnnate as to be present. May the entertainers be as richly blessed as those who were entertained.

